# Mutational and Structural Analysis of Conserved Residues in Ribose-5-Phosphate Isomerase B from *Leishmania donovani*: Role in Substrate Recognition and Conformational Stability

**DOI:** 10.1371/journal.pone.0150764

**Published:** 2016-03-08

**Authors:** Preet Kamal Kaur, Neha Tripathi, Jayesh Desale, Soumya Neelagiri, Shailendra Yadav, Prasad V. Bharatam, Sushma Singh

**Affiliations:** 1 Department of Biotechnology, National Institute of Pharmaceutical Education and Research, SAS Nagar, (Mohali), Punjab, India; 2 Department of Pharmacoinformatics, National Institute of Pharmaceutical Education and Research, SAS Nagar (Mohali), Punjab, India; 3 Department of Medicinal Chemistry, National Institute of Pharmaceutical Education and Research, SAS Nagar (Mohali), Punjab, India; Wake Forest University, UNITED STATES

## Abstract

Ribose-5-phosphate isomerase B from *Leishmania donovani* (*Ld*RpiB) is one of the potential drug targets against visceral leishmaniasis. In the present study, we have targeted several conserved amino acids for mutational analysis (*i*.*e*. Cys69, His11, His102, His138, Asp45, Tyr46, Pro47 and Glu149) to gain crucial insights into their role in substrate binding, catalysis and conformational stability of the enzyme. All the eight *Ld*RpiB variants were cloned, sequenced, expressed and purified. C69S, H102N, D45N and E149A mutants exhibited complete loss of enzyme activity indicating that they are indispensable for the enzyme activity. Kinetic parameters were altered in case of H138N, H11N and P47A variants; however Y46F exhibited similar kinetic behaviour as wild type. All the mutants except H138N exhibited altered protein structure as determined by CD and fluorescence spectral analysis. This data was supported by the atomic level details of the conformational changes and substrate binding using molecular dynamic simulations. *Ld*RpiB also exhibited activity with D-form of various aldose substrates in the order of D-ribose > D-talose > D-allose > D-arabinose. Our study provides insights for better understanding of substrate enzyme interactions which can rationalize the process of drug design against parasite RpiB.

## Introduction

Leishmaniasis is a broad spectrum of diseases which has affected approximately 12 million people worldwide while 350 million people are at risk every year [1]. One of the clinical forms of this disease is visceral leishmaniasis (VL) which is fatal if left untreated. It is caused by *Leishmania donovani* in Indian subcontinent and East Africa whereas, VL is caused by *Leishmania infantum* in Europe, North Africa and Latin America [2]. In the present scenario, the current drugs have limited applications due to inappropriate usage and emergence of resistance towards pentavalent antimonials. Thus, it is mandatory to explore new drug targets by making use of the fundamental biochemical and metabolic disparities between the parasite and host [3].

One such drug target is the type B form of Ribose-5-phosphate isomerase (RpiB) enzyme in *L*. *donovani* which is completely absent in the mammalian host. Rpi is an essential enzyme of pentose phosphate pathway (PPP). The PPP is important for the parasite as it is expected to protect it against free radical produced by the host and is required to synthesize Ribose 5-Phosphate (R5P) for nucleotide synthesis [4]. Rpi has two isoforms *i*.*e*. RpiA and RpiB which are completely unrelated to each other but catalyzes the same reversible aldose-ketose isomerization reaction between R5P and Ribulose-5-phosphate (Ru5P). The essentiality of Rpi enzyme has been observed in *Escherichia coli* which has both RpiA and RpiB forms and double mutant of rpiA^-^/rpiB^-^ resulted in severe impairment of bacterial growth [5]. The silencing of RpiB in *T*. *brucei* by RNAi has resulted in reduced parasite growth and infectivity thus, highlighting the relevance of this enzyme as a drug target [6]. In *L*. *donovani*, RpiB was biochemically and biophysically characterized for the first time from our group [7, 8].

Till date, RpiB structure prediction has been performed for many organisms such as *E*. *coli*, *Mycobacterium tuberculosis*, *Trypanosoma cruzi* and *L*. *major* but interestingly the structures of RpiB from these organisms were very much similar [9–11]. It was proposed that isomerisation of R5P to Ru5P requires the open conformation of R5P (C-form). Structural data of RpiB from *M*. *tuberculosis* and *E*. *coli* reveals that the enzyme first binds to the furanose conformation of R5P (F-form) (S1 Fig) and then catalyzes the ring opening [10, 12–14]. Unlike hydride transfer mechanism as reported for other sugar isomerases such as L-rhamnose isomerase and xylose isomerase, both RpiA and RpiB are reported to act *via* a cis-enediolate intermediate [15, 16]. The active site architecture of RpiA from *Homo sapiens* (*Hs*RpiA) and RpiB from *Leishmania major* (*Lm*RpiB) is highly different. Contrary to the *Hs*RpiA, *Lm*RpiB active site is characterized by the presence of many aromatic amino acid residues (His11, Tyr46, His102 and His138) apart from the negatively charged residue (Asp10 and Cys69) [9]. This significant difference in the catalytic site strengthens the possibility of designing parasite specific inhibitors without affecting the homologous human protein.

The aldose-ketose isomerisation potential of RpiB enzymes has made this enzyme useful for converting many rare monosaccharides to monosaccharide phosphate substrates. Due to its broad substrate specificity, RpiB has been used to convert number of sugars like D-psicose to D-allose, D-talose to D-tagatose, and D-ribose to D-ribulose [17]. It has been observed that RpiB from *Clostridium thermocellum*, *Clostridium difficile* and *Thermotoga maritama* can catalyze the production of various valuable sugars by interconversion of aldoses to ketoses or vice versa and exhibits broad substrate specificity [17–19].

Using site directed mutagenesis approach against the highly conserved residues identified in the RpiB protein of *L*. *donovani*, we set out to identify their role in structural and functional integrity of RpiB. The effect of mutations was also investigated on the conformational state of the protein by CD and fluorescence spectroscopy. *Ld*RpiB and its mutants were also examined for the substrate specificity with other aldose sugars apart from R5P. *In silico* analyses of *Ld*RpiB enzyme and its mutants was done to explore the structural and functional differences among the *Ld*RpiB wild type and its mutants. Using homology modeling, the 3D structures of the enzymes (wild type as well as eight mutants) were generated. Molecular docking studies were performed with two substrate conformations *i*.*e*. F-form and C-form, to gain insights into the active site and catalytic mechanism of the enzyme. Further, the electrostatic surface potential and the binding pocket volumes were analysed to understand the differences among the various mutants and the wild type *Ld*RpiB homology models. Thus, our work provides insights for better understanding of substrate enzyme interactions which can rationalize the process of specific drug design against *Ld*RpiB.

## Materials and Methods

### Materials

*Pfu* DNA polymerase was procured from MBI Fermentas, restriction endonucleases were procured from Genei Bangalore. Gel extraction and plasmid isolation kits, HIS-Select® HF nickel affinity gel column, R5P, Ru5P and D-Ribose were procured from Sigma. Substrates like D-allose, D-psicose, D-talose, D-tagatose and D-arabinose, were procured from Alfa Aesar. All the remaining chemicals used were of analytical grade.

### Construction of the *Ld*RpiB mutants

*Ld*RpiB mutants were generated by overlap extension PCR (OE-PCR) technique [20]. Two sets of primers were designed specific to various mutations for OE-PCR (P1/P4 and P2/P3 primers) (S1 Table). Since, H11N position is at N-terminal of *Ld*RpiB sequence, a long sense primer of 78 bp was designed and used with P2 antisense primer to get a PCR product of 519 bp with H11N mutation. The full length *Ld*RpiB gene with desired mutation was cloned between *Nde*I and *Xho*I restriction sites of pET30a expression vector. The authentication of the *Ld*RpiB mutants was done by automated sequencing (Eurofins Biotech).

### Expression and purification of *Ld*RpiB mutants

Positive clones of *Ld*RpiB mutants were transformed into *E*. *coli* BL21 (DE3) cells and were expressed by induction with 0.1 mM IPTG at 37°C for 3 h, same as wild type *Ld*RpiB protein. The protocol for the purification of *Ld*RpiB mutants by HIS-Select® HF nickel affinity gel column was same as described earlier. The recombinant protein was quantitated using BCA method. The purified samples were loaded on to 12.5% SDS-PAGE to check integrity of the purified protein.

### Enzyme activity and kinetic analysis of *Ld*RpiB mutants

Enzyme activity was determined at 290 nm in Tecan microplate reader. The final reaction mix of 250 μL containing 2.1 μM of recombinant protein in NaH_2_PO_4_ (pH 7.5) and R5P as substrate, was incubated at room temperature and the enzyme activity was measured. Specific activities of wild type *Ld*RpiB and all the mutants were determined using saturating concentrations of the R5P substrate. The results of mutants were expressed relative to the specific activity of the wild type *Ld*RpiB enzyme. For kinetic analysis, various concentrations of R5P (1 mM to 10 mM) were taken. After addition of all reaction components, the samples were scanned for 5 min to observe the change in enzyme activity. The data was fit to Michaelis-Menten equation using a nonlinear regression algorithm. One unit of enzyme activity is defined as the amount of enzyme that will produce 1 nmol of the product per minute.

### Fluorescence spectroscopy

Fluorescence scans of the wild type *Ld*RpiB and its mutants were performed in Varian Cary Eclipse spectrofluorometer using 10 mm pathlength cuvette. The intrinsic tryptophan fluorescence was measured by excitation at 290 nm and the emission spectra was recorded from 300 nm to 500 nm. The excitation and emission slit widths used were 5 nm and 10 nm respectively. The protein concentration of the wild type *Ld*RpiB and its mutants was taken as 1.76 μM in 10 mM NaH_2_ PO_4_ (pH 7.5) buffer for scanning. In order to perform the substrate binding experiments, 10 mM R5P was added to individual mutants and the reaction was carried out at room temperature. The final scan was obtained after an average of three independent scans for each sample.

### Circular dichroism

Secondary structure analysis was performed in Jasco J-815 CD spectrophotometer by using 10 mm cuvette and the scans were recorded from 250 to 190 nm. The background buffer base-line was corrected for each spectrum and each scan was recorded at the speed of 50 nmmin^-1^. The final scan obtained was the average of three independent scans taken. The protein concentration of the wild type *Ld*RpiB and its mutants used in experiment was 0.03 μg/μL in 10 mM sodium phosphate buffer (pH 7.5). The values at θ_222_ nm was considered to evaluate the Mean residue ellipticity (MRE) ‘θ’ *i*.*e*. MRE = θ_obs_ x MRW/10lc. ‘θ’ is CD millidegree, ‘MRW’ is mean residual weight, ‘l’ is pathlength of cuvette and ‘c’ is concentration of the protein.

### Substrate specificity of *Ld*RpiB for aldoses

The specific activity of *L*. *donovani* RpiB for (D-) form of aldoses as well as R5P was determined by the “cysteine carbazole” method. The reaction mixture (160.5 μL) contained 10 mM of sodium phosphate buffer (pH 7.5), 10 μL of the recombinant enzyme at a concentration of 0.33 μM, 10 mM of final substrate concentration, 2% cysteine, 0.12% carbazole, 75% H_2_SO_4_ and 10% of 10 μL of trichloroacetic acid (TCA). The spectrophotometric determination was performed by taking the absorbance at 562 nm. The initial reaction between the enzyme and substrate was carried out for 20 min at room temperature and the reaction was stopped by adding TCA. Cysteine carbazole reagent was added and kept for incubation at 40°C for 30 min. One unit of enzyme activity was defined as the formation of 1 nmole of the ketose sugar within 1 min under the assay condition [21]. Standard curves of various ketoses were plotted by taking concentrations ranging from 1 mM to 20 mM. Molar coefficients were obtained from standard curves of respective ketoses and were used to calculate specific enzyme activity of the wild type *Ld*RpiB and its mutants.

### Statistical analysis

The data is represented as mean ± S.D. of values obtained from indicated number of experiments. Statistical analysis of the data was performed using Graph-Pad Prism software. Differences from groups were tested by ANOVA and were considered to be statistically significant at *p*< 0.05.

### Homology modeling of *Ld*RpiB wild type and mutants

The crystal structure of RpiB from *L*. *donovani* is not available in the Protein Data Bank (PDB). Therefore, homology modeling was used to predict and develop the 3-dimensional structure of the homodimer of the wild type *Ld*RpiB and various mutants. The sequence of the target protein *i*.*e*. *Ld*RpiB was retrieved from UniProtKB database (UniProt accession number: G9JM00). Identification of homologous proteins was performed with the help of BLAST server by search against the structural database PDB. After a thorough analysis, RpiB from *T*. *cruzi* (*Tc*RpiB) complexed with R5P (PDB ID: 3K7S, resolution: 1.90 Å) was selected as template (sequence identity 49%; sequence similarity 66%; E-value 4e^-47^) [11]. The sequence alignment between the template and the target sequence was performed using ClustalW. The Modeller 9.10 software was used to generate the homology models of wild type *Ld*RpiB [22]. The generated models were evaluated through SAVES server (http://services.mbi.ucla.edu/SAVES/) by calculating Errat score, Ramachandran Plot score, Z-score etc. Loop optimisation was further performed using Modeller 9.10 to improve the structural quality of the models. For mutational analysis of *Ld*RpiB, mutations were inserted in the prepared homology model with the help of Build Mutant utility of Accelarys Discovery Studio 2.5. Their corresponding 3D structures were employed for further molecular modeling analysis. As the water molecules in the binding pocket are very important for substrate recognition and binding to the enzyme, therefore coordinates of seven co-crystalized water molecules, which showed interactions with the substrate in the crystal structure of *Tc*RpiB (W176, W180, W195, W204, W222 and W427) were modelled in the prepared homology models of wild type as well as the mutants and were renumbered from W1-W7 for simplicity.

### Molecular docking studies

The 3D structures of the small molecules considered in this study (F-form and C-form of R5P) were prepared using build module of Maestro 9.3 [(Maestro (version 9.3). New York: Schrödinger, LLC; 2012)] after assigning appropriate charges. The LigPrep module (Maestro 9.3) was used to generate the low energy tautomeric, ionization and stereoisomeric states at physiological pH (7.0±2.0). The macromolecular structures were prepared using Protein Preparation Wizard of Maestro 9.3 by adding the missing hydrogens, assigning the right bond order and optimizing the orientations of hydroxy group (in Ser, Thr and Tyr), amino group (in Asn and Gln) and ionization state (His) [23]. The water molecules modeled from the crystal structure were maintained for the molecular docking studies. The receptor interaction grid was generated at the centroid of the bound ligand in crystal structure of *Tc*RpiB, PDB ID: 3K7S (grid center: 16.29, 38.86, 36.45) and reported active site residues in the homology models (grid center: 16.23, 38.88, 36.43). The grid box was extended up to 10 Å as the innerbox and 20 Å as the outer box covering the binding site cavity completely. The prepared ligands were docked in the generated grids in the *Tc*RpiB and various homology models using Glide docking module available in Maestro 9.3. Each of the poses were analysed for RMSD with the co-crystalized ligand, molecular recognition interactions and Glide docking score [24].

### Molecular Dynamics Simulation

Molecular dynamics simulation studies were performed on the substrate-enzyme complexes generated from the molecular docking studies using AMBER 11 package [25]. The partial atomic charges were assigned using Restrained Electrostatic Potential (RESP) method of *antechamber* module of AMBER [26]. For the preparation of ligands and protein, General Amber Force Field (GAFF) and Amber ff99SB force field were implemented respectively [27, 28]. System (enzyme-substrate complex) was solvated using TIP3P water, with solvation box extended to 12 Å in each direction of the solute forming an octahedral box that can mimic an infinite system in MD simulations [29]. After an initial minimisation of the system, gradual heating of the system from 0 to 300K was performed with system restraint of 2 kcal/mol/Å^2^ under NVT ensemble for 50 ps. The density equilibration of the protein was carried out under NPT ensemble in three phases *i*.*e*. 50 ps equilibration with weak restraint of 2 kcal/mol/Å^2^, followed by 50 ps with restraint of 1 kcal/mol/Å^2^ and then unrestrained density equilibration for 100 ps. The constant pressure equilibration (NPT) of 1 ns was performed at 300 K and 1 atm pressure (pressure relaxation time of 2.0 ps). Finally, production run for 5 ns was performed under NPT ensemble during which cut-off distance of 12 Å was kept for calculating the non-bonded interactions and long-range electrostatic interactions were treated with the Particle-Mesh Ewald (PME) method [30]. The calculation of relative binding free energy for the protein-ligand complex formation was performed using Molecular Mechanics-Generalized Born Surface Area (MM-GBSA) method available in Amber 11 package keeping all other parameters at their default values [31]. The calculations were performed on the last 2 ns trajectories obtained from MD simulations of protein-ligand complexes to ensure good conformational sampling and reliable binding free energy calculations.

## Results and Discussion

### Sequence alignment and generation of the *Ld*RpiB mutants

**Fig 1:**
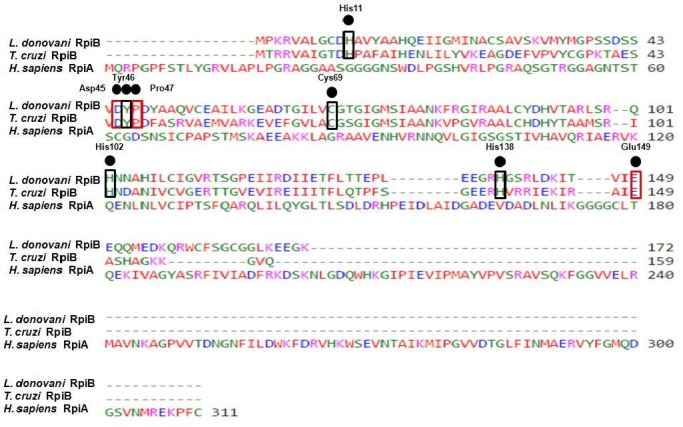
Sequence alignment of *Ld*RpiB amino acid sequence with amino acid sequence of *T*. *cruzi* RpiB and *H*. *sapiens* RpiA using Clustal W. Black dot represents the amino acids targeted for site directed mutagenesis, black box represents the amino acids present in the substrate binding pocket of *T*. *cruzi* and red box represents the other conserved amino acids.

*L*. *donovani* RpiB amino acid sequence was aligned with the amino acid sequence of *T*. *cruzi* RpiB and human RpiA sequence. *Ld*RpiB showed 49% identity and 66% similarity with *Tc*RpiB whereas with *Hs*RpiA, *Ld*RpiB showed 16% identity and 30.4% similarity. The highly conserved amino acids between the RpiB’s manifest the probability of these amino acids to be part of functionally and structurally important domains. The amino acid residues targeted for site directed mutagenesis in the present study are marked as black dot (Fig 1). The residues present in the substrate binding pocket in *T*. *cruzi* are in black box (His11, Tyr46, Cys69, His102, and His138) and other conserved residues are highlighted in red box (Asp45, Pro47 and Glu149). The corresponding residues in the human RpiA (functionally homologous enzyme) are non-identical and thus, indicate the suitability of *Ld*RpiB for antileishmanial drug design. All the eight *Ld*RpiB mutants were generated and confirmed by automated sequencing. SDS-PAGE analysis of mutants confirmed that all the eight *Ld*RpiB mutants possessed the same subunit size ∼19 kDa (data not shown).

### Effect of amino acid mutations on substrate binding and kinetic parameters of *Ld*RpiB

**Fig 2:**
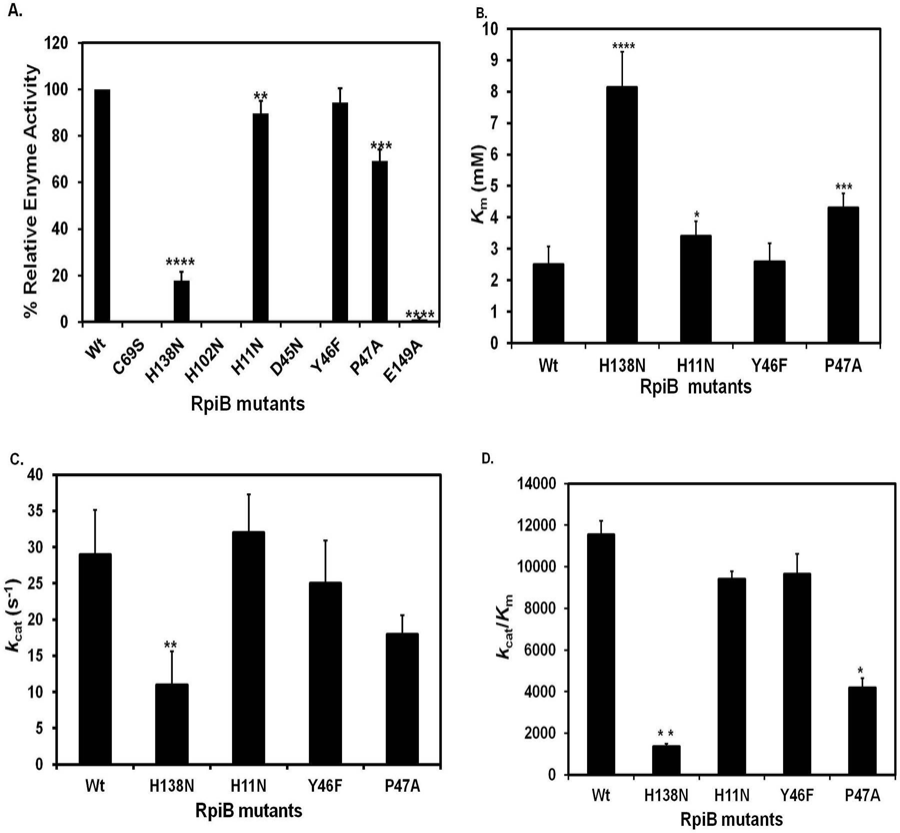
Biochemical analysis of *Ld*RpiB wild type (wt) and various mutants. (A) Percentage relative enzyme activity. Kinetic parameters of *Ld*RpiB wild type (wt) and various mutants. (B) A graph plot of *K*_m_ values for wild type *Ld*RpiB and its mutants. (C) A graph plot of *k*_cat_ values for *Ld*RpiB wt and its mutants and (D) A graph plot of *k*_cat_ /*K*_m_ values for *Ld*RpiB wt and its mutants. Data represents the mean ± S.D. from three experiments performed in duplicates, * represents *p* ≤ 0.05, ** represents *p* ≤ 0.01 and *** represents *p* ≤ 0.001.

R5P was used as a substrate to examine the reaction specificity and kinetic parameters [Michaelis constant (*K*_m_), turnover number (*k*_cat_) and catalytic efficiency (*k*_cat_ / *K*_m_)] of the wild type *Ld*RpiB and its mutants. Table 1 depicts the values of kinetic data obtained for the wild type *Ld*RpiB and its mutants. The relative enzyme activity was calculated where C69S, H102N, D45N and E149A mutants did not show any activity whereas, in case of the mutants H138N, H11N, P47A and Y46F, there was almost 82.2%, 10.7%, 30.7% and 5.6% loss in enzyme activity respectively (Fig 2A). Hence, no kinetic parameters could be calculated for the mutants C69S, H102N, D45N and E149A which were found to be indispensable for the enzyme activity. In *T*. *cruzi*, the final isomerisation step is catalyzed by the charged C69 residue (Cys66 in *E*. *coli*) which forms the catalytic base and accepts the proton from C2 of the substrate and Thr71 transfers H^+^ from O_2_ to O_1_ forming enediolate intermediate state. Additionally, NH groups of residues Gly70 to Gly74 aid in stabilizing the negative charge of intermediate and finally, the Cys69 returns the proton to C1 resulting in R5P [11, 14]. The enzyme was also reported to be completely inactive with C69A mutation in *Tc*RpiB suggesting its importance in enzyme catalysis [21]. In *Mt*RpiB, Glu75 forms the catalytic base instead of Cys which is responsible for transferring a proton between C1 and C2 as observed for Cys66 in *Ec*RpiB [10, 32, 33]. H102 has been proposed to donate a proton to ring oxygen atom O4 whereas, H138 would act as a base that accepts a proton from O1H in *M*. *tuberculosis* [33]. In *T*. *cruzi* H102A mutant of RpiB has shown 10 fold decrease in *k*_cat_ with no change in *K*_m_ value, indicating the role of H102 in catalysis [21]. The *Ec*RpiB H99N mutant (His102 in *L*. *donovani*) exhibited a *K*_m_ value similar to the wild type enzyme but the *k*_cat_ value was reduced by 26 fold [14].

**Table 1:** Kinetic parameters for wild type *Ld*RpiB and its mutants.

	*K*_m_ (mM)	*V*_max_ (Umg^-1^min^-1^)	*k*_cat_ (s^-1^)	*k*_cat_ / *K*_m_ (M^-1^ s^-1^)
RpiB (Wt)	2.51 ± 0.56	0.423 ± 0.089	29 ± 6.1	11175.48 ± 3057.81
C69S	ND	ND	ND	ND
H102N	ND	ND	ND	ND
H11N	3.40 ± 0.47	0.257 ± 0.037	32 ± 5.3	9512.02 ± 2249.08
H138N	8.14 ± 1.13	0.364 ± 0.098	11 ± 4.6	1355.07 ± 365.87
E149A	ND	ND	ND	ND
D45N	ND	ND	ND	ND
Y46F	2.59 ± 0.59	0.401 ± 0.074	25 ± 5.9	9820.05 ± 2918.75
P47A	4.30 ± 0.46	0.294 ± 0.065	18 ± 2.6	4241.06 ± 1274.27

ND, not determined.

**Fig 3:**
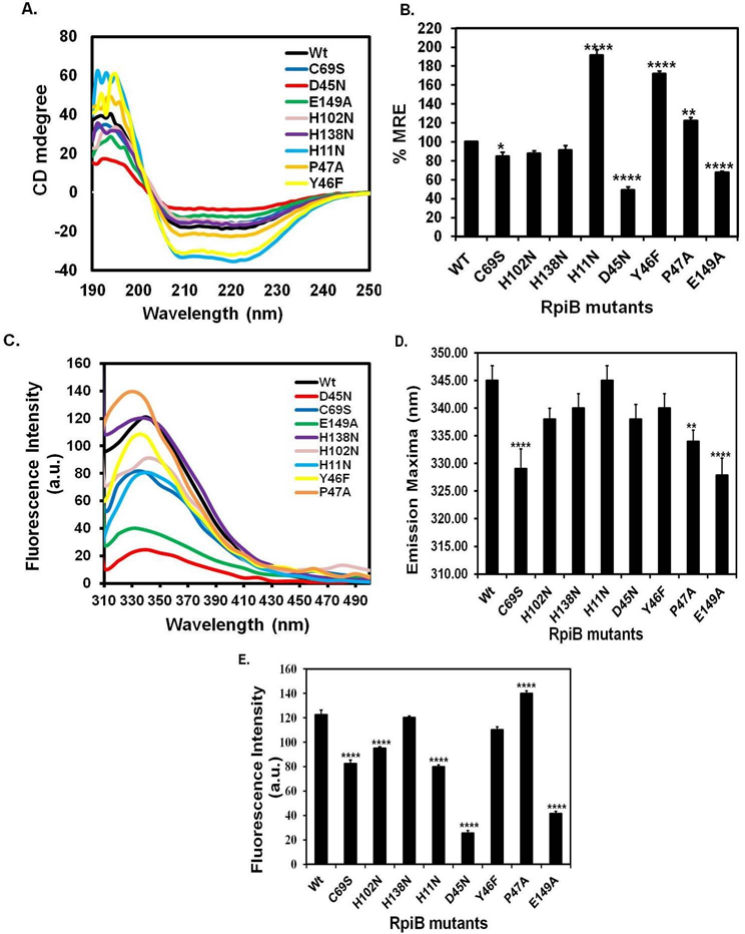
Biophysical analysis of *Ld*RpiB wild type (wt) and various mutants. (A) CD spectra of *Ld*RpiB and its mutants from 190–250 nm. (B) Changes in percentage mean residue ellipticity of *Ld*RpiB and its mutants. Data represents the mean ± S.D. from three experiments performed in duplicates, * represents *p* ≤ 0.05, ** represents *p* ≤ 0.01 and *** represents *p* ≤ 0.001. (C) Fluorescence Emission spectra at 290 nm wavelength. (D) and (E) Changes observed in the emission maxima and fluorescence intensity of wild type RpiB and its mutants respectively. Data represents the mean ± S.D. from three experiments performed in duplicates, * represents *p* ≤ 0.05, ** represents *p* ≤ 0.01 and *** represents *p* ≤ 0.001.

His138Asn mutation in H138N led to increase in *K*_m_ value by 3.2 fold (*p*< 0.0001) with significant decrease in the *k*_cat_ value (2.6 fold) and 8.5 fold decrease in *k*_cat_ /*K*_m_ value as compared to wild type *Ld*RpiB (Figs 2B–3D). However, in *T*. *cruzi*, *K*_m_ values were doubled in case of H138A mutant without affecting *k*_cat_ values, thus suggesting its role in substrate binding but not in ring opening [21]. In case of *M*. *tuberculosis* RpiB, His138 was suggested to play a role in furanose ring opening as analysed by X- Ray structures [21, 33]. In contrast, H11N *Ld*RpiB mutant showed 1.3 fold and 1.1 fold increase in *K*_m_ and *k*_cat_ values. In *T*. *cruzi*, H11A mutation resulted in 6 fold higher *K*_m_ value compared to the wild type enzyme. This suggested its role in the stability of the enzyme rather than enzyme catalysis [21]. Structural analysis has shown that His11 in *T*. *cruzi* and His10 in *E*.*coli* interact with the phosphate moiety of the ligand [10, 11]. Since, the nature of the mutated residue (Tyr46Phe) was same *i*.*e*. aromatic therefore, there was no significant change in the enzyme activity. For P47A mutant, *K*_m_ value was doubled and there was 2.7 fold decrease in the catalytic efficiency. Though, P47 is not reported in active site in *T*. *cruzi or L*. *major*, yet it has a role in alteration of the enzyme activity. The mutation of these targeted amino acids suggests the role of the conserved residues in substrate recognition as well as in enzyme catalysis.

### CD spectra of *Ld*RpiB and its mutants

Far UV-CD spectra were measured for the recombinant wild type *Ld*RpiB and its mutants (Fig 3A and 3B). Analysis of CD signal at 222 nm revealed that all the mutants (except H102N and H138N) had significantly altered conformational stability of the enzyme. In C69S mutant, there was almost 15% loss of α-helical content with respect to wild type. This probably led to loss of active site architecture and thus rendered enzyme inactive. Apart from this, D45N and E149A mutants also showed significant loss of helical content as the % MRE at 222 nm decreased by 50% and 32.4% respectively in comparison to the wild type. The polarity of negatively charged residues has also changed in D45N and E149A mutants which probably hampered ionic interactions required for secondary structure stabilization. Surprisingly, H11N, Y46F and P47A mutants displayed increased % MRE compared to wild type *Ld*RpiB (*i*.*e*. 91.1%, 71.15% and 22.5% respectively). This signifies the participation of these mutants in conformational changes of *Ld*RpiB enzyme. Hence, Cys69, Asp45, Glu149, Pro47 and His11 are residues which are involved both in enzyme activity and maintenance of the structural integrity. The H102N and H138N mutations did not alter the secondary structure of the enzyme but both are involved in substrate recognition and enzyme catalysis.

### Intrinsic fluorescence of wild type *Ld*RpiB and its mutants

The effect of mutations on tertiary structure of the recombinant *Ld*RpiB protein was also monitored by fluorescence spectroscopy (Fig 3C–3E). The wild type *Ld*RpiB showed emission maxima at 344 nm on excitation at 290 nm. The C69S mutant showed significant changes in fluorescence spectra with blue shift of 15 nm and quenched fluorescence intensity (*i*.*e*. 57% of wild type). E149A mutant also showed significant alteration in tertiary content of the enzyme leading to blue shift (17 nm) and quenching of fluorescence intensity (*i*.*e*. 34% of wild type). Interestingly, the mutants H102N, H11N and D45N did not show any change in emission maxima but a significant quenching of fluorescence was observed. P47A exhibited blue shift (11 nm) in emission maxima; however, there was also an increase in overall fluorescence intensity (by 14%) in comparison to wild type *Ld*RpiB enzyme. The H138N and Y46F mutants did not show any alteration in tertiary structure of enzyme and were found to be similar to the wild type. Hence, except H138N and Y46F mutants, all other *Ld*RpiB mutants exhibited significant alteration in the structural conformation of the enzyme.

### Substrate specificity of wild type *Ld*RpiB and its mutants

**Fig 4:**
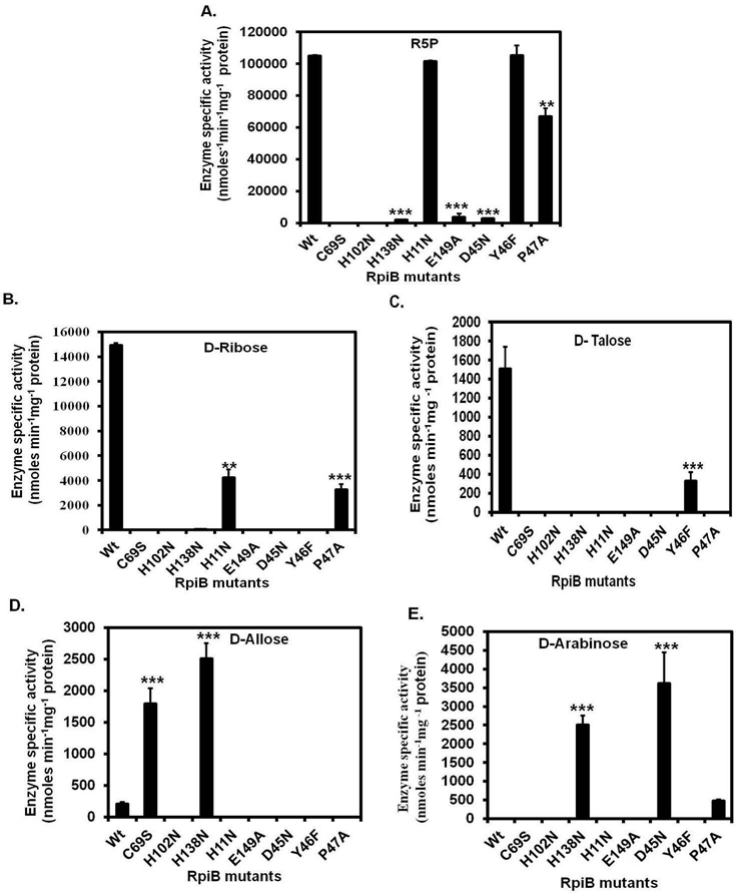
Substrate specificity of *Ld*RpiB using D- form of aldose sugars. (A) R5P, (B) D- Ribose, (C) D- Arabinose, (D) D- Talose and (E) D- Allose. Data represents the mean ± S.D. from three experiments performed in duplicates, * represents *p* ≤ 0.05, ** represents *p* ≤ 0.01 and *** represents *p* ≤ 0.001.

The recombinant *Ld*RpiB enzyme exhibited maximum activity with R5P, as estimated by colorimetric method and mutants like H11N and Y46F exhibited comparable enzyme activities as wild type (Fig 4A). P47A mutant retained approximately 70% of enzyme activity with R5P. C69S and H102N mutants became completely inactive and E149A, D45N and H138N mutants showed significant decrease in enzyme activity with R5P (Fig 4A). Surprisingly, *Ld*RpiB showed maximum enzyme activity with D-ribose after R5P though the activity was 7 fold lower than R5P. Hence, the wild type *Ld*RpiB exhibited specificity with aldose substrates in the order D-ribose > D-talose > D-allose > D-arabinose. Thus, D- ribose can also be used to design structural analogues as *Ld*RpiB inhibitors and aid in the development of new antileishmanial agents. The RpiB’s from other organisms like *C*. *thermocellum* has shown maximum activity with L-talose apart from sugar phosphates. *C*. *difficile* RpiB exhibited activity only with aldose substrates such as D-ribose, D-allose, L-tallose, L-lyxose, D-gulose and L-mannose, [17–19] whereas, RpiB from *T*. *maritima* showed substrate specificity for both D- and L- form of ribose, allose and talose [19].

It was interesting to observe that *Ld*RpiB mutants which were inactive against R5P, exhibited substrate specificity towards other aldose substrates except H102N and E149A. Interestingly, C69S mutant was active with D-allose and D45N was active with D-arabinose. H138N mutant (which was less active when R5P was used as substrate) showed specificity towards D-arabinose and D-allose. On the other hand, H11N mutant exhibited 2.6 fold decrease in activity with D-ribose when compared to R5P and was inactive with other substrates. P47A mutant displayed enzyme activity with two aldoses *i*.*e*. D-ribose and D-arabinose. Y46F mutant which showed similar activity like wild type with R5P, also showed specificity towards D-talose though the activity decreased by 4 fold in comparison to wild type *Ld*RpiB. Thus, it can be observed that mutations have altered the specificity of the *Ld*RpiB enzyme towards various aldose substrates (Fig 4B–4E).

### Analysis of structural changes in wild type *Ld*RpiB and its mutants upon R5P binding

**Fig 5:**
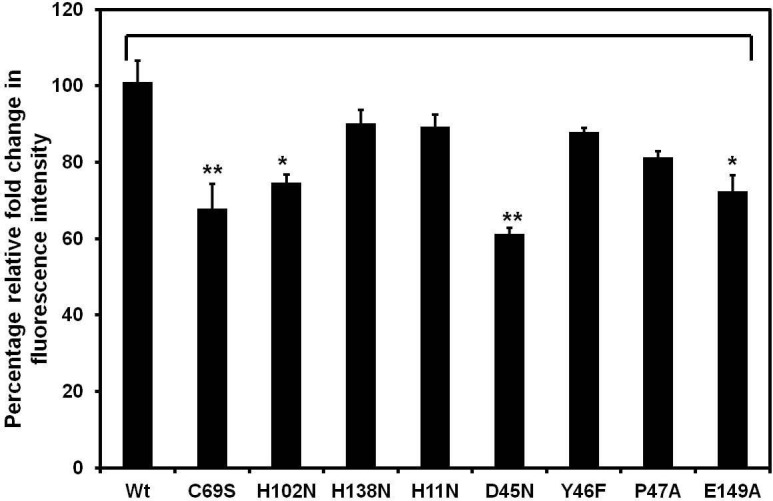
A graph plot of percentage relative fold change in fluorescence intensity of *Ld*RpiB wild type and its mutants. Data represents the mean ± S.D. from three experiments performed in duplicates, * represents *p* ≤ 0.05 and ** represents *p* ≤ 0.01.

The phenomenon of binding of R5P substrate to the recombinant *Ld*RpiB wild type and its mutants can be studied by fluorescence spectroscopy (Fig 5). When R5P was allowed to react with *Ld*RpiB wild type, there was quenching of the fluorescence which depicted that there is a significant conformational change in the enzyme upon substrate binding. There was almost 1.6 fold decrease in the fluorescence intensity of the wild type upon R5P binding and this fold change was considered as 100% and hence, the relative fold change was calculated for *Ld*RpiB mutants with respect to *Ld*RpiB wild type. In case of inactive mutants *i*.*e*. C69S, H102N, D45N and E149N, there was (approximately 33.1%, 26.2%, 39.7% and 28.6% respectively) reduction in fold changes of these mutants in comparison to the wild type. This indicated that R5P was unable to bind with the enzyme possibly due to distortion of active site upon these mutations which rendered enzyme inactive. On the other hand, active mutants (H11N, Y46F, P47A and H138N) displayed similar fluorescence spectra (closer fold change values) comparable to *Ld*RpiB wild type. The active mutants H11N, Y46F, P47A and H138N retained 89.4%, 87.9%, 81.3% and 90.1% respectively fold change upon R5P binding with respect to *Ld*RpiB wild type. This indicated that in case of the active mutants upon R5P binding there was no alteration in the substrate binding site which allowed the essential substrate enzyme interactions required for isomerisation reaction.

### Three dimensional structure prediction of *Ld*RpiB by homology modeling

In order to provide insights into the *in vitro* results, the homology models for the wild type and various mutants of *Ld*RpiB were prepared. The various validation parameters such as Ramachandran plot, Errat plot score etc. suggested acceptability of the prepared models (Table 2). The Root Mean Square Deviation (RMSD) between template and *Ld*RpiB wild type model was calculated to be 0.087 Å which suggests good quality of the model. Further, the active site residues were almost overlapping in the two proteins (S2 Fig). The residues His11, Asp10, Cys69, His102, Tyr46, His138 were found to lie in the active site of *Ld*RpiB homology model similar to *T*. *cruzi* and the predicted residues of *L*. *major* [9, 11]. Apart from these active site residues, three other conserved residues (Asp45, Pro47 and Glu149) were also evaluated for their role in structural integrity and catalytic function of *Ld*RpiB.

**Table 2:** Homology model validation parameters for template, *Ld*RpiB wild type and various mutants.

		Ramachandran Plot for All Residues (%)			
Serial number	Mutants	Favoured	Allowed	Disallowed	Errat Plot Score (in %)
1	*Tc*RpiB	98.70	1.30	0.00	98.61
2	*Ld*RpiB (WT)	97.80	2.20	0.00	92.88
3	*Ld*RpiB (C69S)	98.00	2.00	0.00	92.88
4	*Ld*RpiB (D45N)	97.80	2.20	0.00	92.19
5	*Ld*RpiB (H11N)	97.80	2.20	0.00	91.84
6	*Ld*RpiB (H102N)	97.80	2.20	0.00	92.88
7	*Ld*RpiB (H138N)	97.80	2.20	0.00	92.88
8	*Ld*RpiB (E149A)	97.80	2.20	0.00	92.71
9	*Ld*RpiB (P47A)	97.80	2.20	0.00	93.75
10	*Ld*RpiB (Y46F)	97.80	2.20	0.00	93.40

### Molecular docking and dynamics of substrate in wild type and mutant homology models

**Fig 6:**
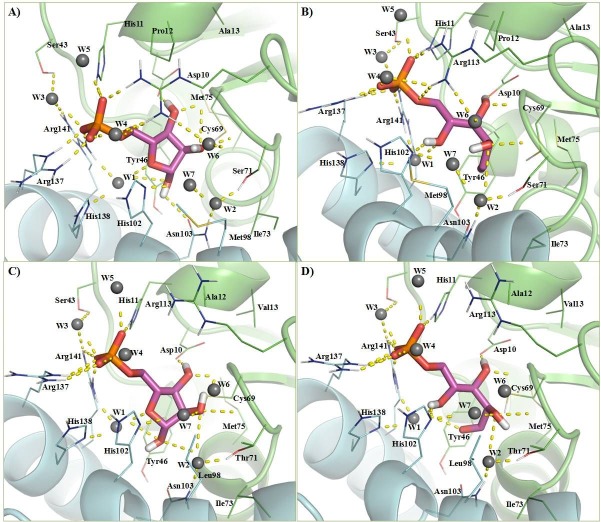
**3D molecular recognition interaction diagram for F-form and C-form of substrate in *Tc*RpiB (A) and (B) and *Ld*RpiB (C) and (D)**.

**Fig 7:**
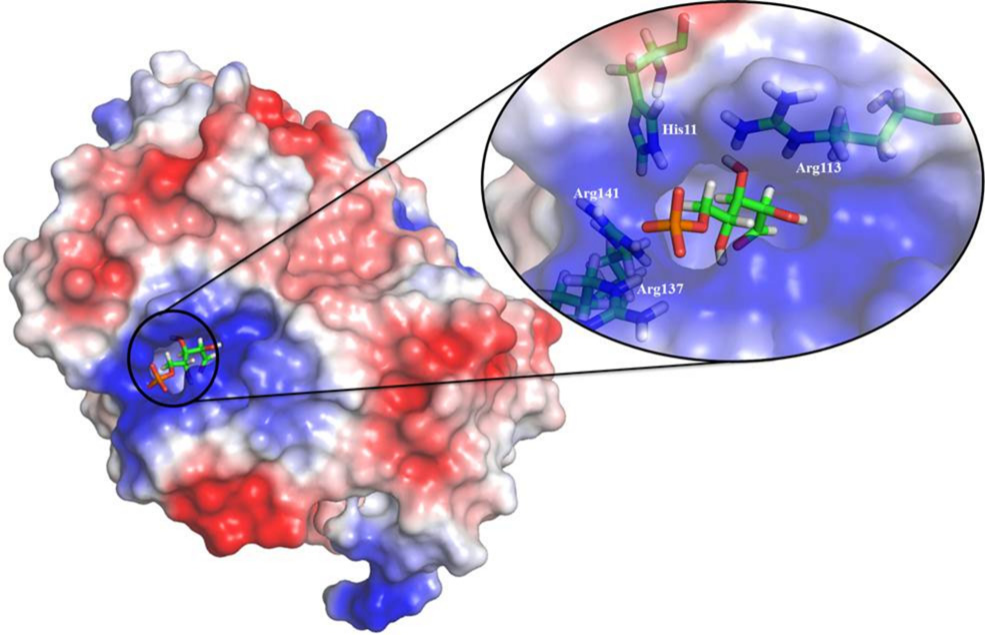
Surface electrostatic potential of *Ld*RpiB. The residues shown in inset are responsible for interaction with the substrate PO_4_^2-^ moiety.

To understand the molecular recognition interactions of the enzyme-R5P complexes, the molecular docking technique is adopted in this work. To validate the protocol, redocking of the C-form of R5P was carried out in the *Tc*RpiB crystal structure. The pose adopted by the substrate after molecular docking and that in the crystal structure are quite similar with an RMSD of less than 0.5 Å (S3 Fig) confirming the acceptability of the adopted protocol. In the next step, molecular docking of the F-form and C-form of R5P has been carried with the *Ld*RpiB wild type and various mutant homology models, and a total of twenty enzyme-substrate complexes were generated. The results from the molecular docking studies are shown in S2 Table. The molecular recognition interactions of F-form and C-form of substrate in *Tc*RpiB and *Ld*RpiB are shown in Fig 6 and S4 Fig. The electrostatic potential surface analysis revealed that nearly 90% of the binding pocket was electropositive due to the presence of basic amino acids (Fig 7). There were three Arg and three His residues which made the active site electropositive towards the surface and Tyr46, Ile73, Gly70, Gly74 etc., which were deep inside the pocket provided an almost electroneutral environment. The basic amino acid residues of the active site which were near the surface of the enzyme interacted with the phosphate (PO_4_^2-^) group of the substrate.

There was no clear distinction in the molecular docking scores of the substrate in various mutants and the pose adopted by C-form and F-form of R5P in the active site were almost identical in all the mutants and the wild type. The reason for this can be attributed to the algorithm used for mutant building in which the backbone was kept fixed and rotamer search for the side chain of the mutated residue was performed which did not affect the environment of the secondary structure. Therefore, molecular dynamics simulation studies were undertaken to generate the optimized structures. All the twenty enzyme-substrate complexes (enzyme: *Tc*RpiB crystal structure, homology model of *Ld*RpiB wild type and the eight *Ld*RpiB mutants; substrate: C-form and F-form of R5P) generated in the molecular docking studies were subjected to molecular dynamics simulations for analysing the stability of intermolecular interactions under dynamical conditions and for calculation of the binding free energies.

**Fig 8:**
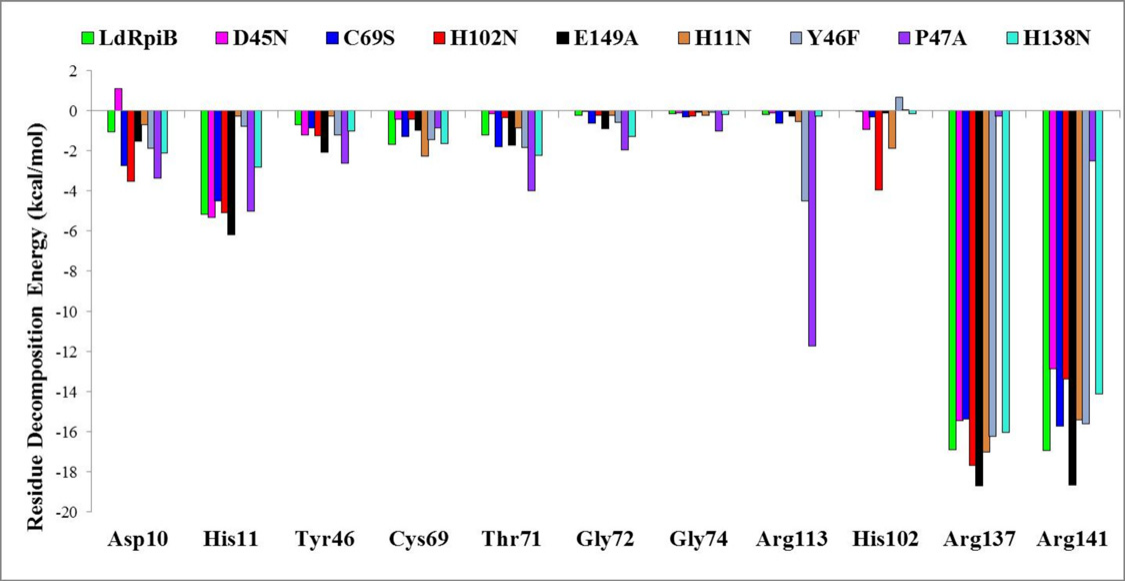
Residue decomposition analysis (GBSA) of residues (for C-form of R5P binding) showing differences among various mutants. The corresponding data for the F-form conformation is provided in S7 Fig (supporting information).

The binding free energy averaged over the last 2 ns trajectory for all the binary complexes are shown in S3 Table). Comparative analysis of hydrogen bond occupancies (S5 Fig) and the residue wise decomposition energy values (Fig 8) for various C-form of substrate and enzyme complexes revealed important facts about the various *Ld*RpiB mutants. The prominent hydrogen bonding interactions with the C-form in the wild type *Ld*RpiB were formed with the ligand *via* Asp10 (126.6%), His11 (32.2%), Thr71 (31.2%), Arg137 (47.0%) and Arg141 (82.8%) (S5 Fig). The hydrogen bonds and electrostatic interactions between PO_4_^2-^ unit of substrate and His11, Arg137 and Arg141 anchored the phosphate group of the substrate in position which allowed catalysis to proceed. The higher hydrogen bond occupancy and residue decomposition energy of Arg137 and Arg141 is due to the interaction of these positively charged residues with the negatively charged PO_4_^2-^ moiety of substrate. The stability of Arg-PO_4_^2-^ electrostatic bond further enhances the strength of the substrate-enzyme complexes [34]. All the eight mutants can be categorised into active mutants (H11N, Y46F, P47A and H138N) and the inactive mutants (D45N, C69S, H102N and E149A). A detailed correlative description on the functional and structural properties of individual mutants, based on *in vitro* and *in silico* results elucidated the basis of activity/inactivity of the enzyme.

### Structural analysis of active mutants

According to the *in vitro* results, the mutants H11N, Y46F, P47A and H138N showed detectable activity in the R5P isomerisation reaction. The analysis of hydrogen bond occupancies of these mutants (from the molecular dynamics data) indicated the presence of 4 crucial bonds with Asp10, Arg137, Arg141 and His11 (with F-form) or Thr71 (with C-form) in all the four mutants (S5 and S6 Figs). A detailed examination of the results is propounded in the individual sub-sections of these mutants.

#### H11N

The mutant H11N is catalytically active with reaction kinetic properties equivalent to the wild type RpiB (1.3 fold and 1.1 fold increase in *K*_m_ and *k*_cat_ values respectively). The H11N mutation marginally reduces the interaction with PO_4_^2-^ unit of R5P, however, the presence of Arg residues compensate for the lost interaction via minor conformational change. This does not affect the R5P binding efficiency with the H11N mutant.

#### Y46F

It was interesting to note that Tyr46 which is found to be present in the substrate binding pocket of *Ld*RpiB and similarly reported for *Tc*RpiB and *Lm*RpiB, did not affect the kinetic parameters [9, 11]. This mutant exhibited similar kinetic behaviour as the wild type and a structural conformation different from the wild type. The hydrogen bond occupancy analysis and per-residue decomposition analysis revealed similar patterns in wild type and Y46F mutant. Therefore, this mutation did not lead to any loss in the functional characteristics of the enzyme. A slight difference in the side chain polarity (-OH group present in Tyr but absent in Phe) can be the cause for the observed structural differences from the wild type.

#### P47A

The mutant P47A was found to be functionally active and structurally different from the wild type. The hydrogen bond occupancies indicated the presence of all the crucial hydrogen bonds but with reduced occupancies. In terms of per-residue decomposition energy contribution, there was a compensation for the Arg141 by Arg113. Therefore, the enzyme activity of P47A was reduced but not completely lost. The reason behind structural differences from the wild type can be attributed to the fact that mutation P47A was huge in terms of steric factors. The sterically rigid side chain of Pro was replaced by flexible side chain of Ala, which led to the changes in the structural conformation of the enzyme.

#### H138N

The *in vitro* results showed that there was loss of activity by 82.2% without any effect on the secondary structure content of the enzyme in case of the His138Asn mutant. His138 was reported to be a part of the catalytic machinery for the ring opening and isomerization reaction [21, 33]. The per-residue decomposition energy values were almost similar to the wild type. Moreover, the crucial hydrogen bonds required for substrate interactions were also present in this mutant and therefore, the activity was not completely lost. However, the hydrogen bond occupancy analysis showed the presence of an intermittent hydrogen bond between C-form and Tyr46 which is located opposite to Asn138 in case of the H138N mutant (S5 Fig). This is caused by the pull of substrate towards Tyr46 (and thus increased distance from Asn138) due to reduced nucleophilic character of Asn138. All these factors have probably led to reduction in the enzyme activity.

### Structural analysis of inactive mutants

The mutants C69S, D45N, H102N and E149A were found to be inactive in the *in vitro* reaction catalysis. The possible reason for this can be explained from binding free energy, hydrogen bond occupancy analysis and the residue wise decomposition energy analysis. The absence of any crucial hydrogen bond can lead to the inactivation of the enzyme. Further, change in the physicochemical properties of amino acid can lead to drastic conformational alterations in the protein structure leading to inactivation of the enzyme. The details are elaborated below.

#### C69S

The mutant C69S was reported to be completely inactive in the reaction catalysis studies and is structurally different from the wild type. The reason is attributed to the change in side chain polarity. In the C69S mutant, the Thr71 H-bond occupancy with C-form is reduced dramatically (from 31.2% to 5.6%). The sulfur atom of Cys is comparatively less electronegative than hydroxyl oxygen of Ser. In the wild type, Cys69 is oriented in a position not facilitating any hydrogen bond between the C-form tail hydroxyl group and the Cys69 side chain. Surprisingly, in C69S mutant, the Ser69 oriented itself in a conformation forming intermittent hydrogen bond with the substrate (S5 Fig) leading to the structural variations from the wild type. The presence of Ser69 and Thr71 side by side is thus, weakening the interaction of Thr71 with the substrate and causing the inactivity of the enzyme.

#### D45N

This mutant completely inactive in terms of catalytic activity. This is supported by the fact that there was an absence of crucial hydrogen bond between the C-form and Asp10 (S5 Fig). Further, S3 Table shows that there was reduced binding free energy of the substrate (-40.39± 5.09 in wild type and -24.95 ± 4.80 in D45N mutant). The residue decomposition analysis showed a positive value of energy contribution in substrate binding for Asp10 in D45N mutant. All these factors provide an explanation to the inactivity of the enzyme as well as the conformational differences with the wild type.

#### H102N

The H102N mutant was also found to be inactive with altered tertiary structure as evident from *in vitro* data. In the H102N mutant, the crucial H-bond between Thr71 and C-form of the substrate is absent leading to inactivity of the enzyme. The presence of hydrogen bond with Asn102 (S5 Fig) and its higher contribution in terms of energy (Fig 8) leading to displaced substrate from the original position are the other differentiating characteristics of this mutant with respect to the wild type. Further, with the F-form of substrate, hydrogen bond with His11 is absent in H102N mutant (S6 Fig). The absence of any structural change in this mutant is due to the similar steric properties of the two amino acids.

#### E149A

The E149A mutant was also found to be inactive in the *in vitro* experimental studies. A lower hydrogen bond occupancy with Asp10 in C-form and higher per-residue energy contribution to the complex formation with the F-form of substrate (probably trapping the enzyme at the ring opening stage) may be the responsible factors for the loss in activity of this mutant (S7 Fig). The comparative RMSD analysis of Glu149 in wild type and Ala149 in E149A mutant showed a higher fluctuation in mutant structure, due to increased flexibility of the helix containing Ala149. These factors are probably leading to the loss of activity and structural variations.

In humans, RpiA form of the enzyme is found to be active in catalyzing the isomerization of R5P to Ru5P whereas, in Leishmania parasite RpiB enzyme plays this pivotal role. *Ld*RpiB amino acid sequence has only 16% identity and 30.4% similarity in comparison to *Hs*RpiA amino acid sequence. In our study, three dimensional structure of *Ld*RpiB enzyme has been predicted which has highlighted the various active site amino acid residues and these are far different from those predicted for *Hs*RpiA from the earlier studies [9]. Structural analysis has shown that *Ld*RpiB enzyme forms hydrogen bond interactions with the open chain form of the substrate with Asp10, His11, Thr71, Arg137 and Arg141. Moreover, His11, Arg137 and Arg141 residues also anchored the phosphate group of the substrate in a position to precede catalysis. In case of *Hs*RpiA, K82, S106, T107 and K200 residues were found to interact with the phosphate group of the substrate [9]. In *Ld*RpiB, C69 residue forms the catalytic base and accepts the proton from C2 of the substrate and T71 transfers H^+^ from O2 to O1 forming enediolate intermediate. Whereas, in *Hs*RpiA D160 and E182 as well as water molecules aids the catalytic mechanism. D160 acts as a base by removing one proton from O1 of R5P whereas; the water molecule donates H^+^ to O4. On the other hand, E182 catalyzes the opening of furanose ring by removing H^+^ from O1 and water molecule also participates in furanose ring opening by donating a proton to O4 of R5P [9]. *Ld*RpiB has also shown the presence of many aromatic and basic amino acid residues (Y46, H11, H102 and H138) which are absent in *Hs*RpiA.

This study has revealed a number of important facts about the *Ld*RpiB enzyme which can be used to direct the anti-leishmanial drug design. The highly electropositive nature of the substrate binding cavity is identified using the molecular modeling techniques. Further, possible structural dynamics can be speculated to be involved in substrate binding in the active site due to the highly electropositive potential. The molecular recognition interactions required for the substrate-enzyme binding were explored. The presence of a terminal electronegative group and small molecular size of ligand are recognized as important pharmacophoric features to inhibit the *Ld*RpiB. These outcomes can be used to identify the potential hits against *Ld*RpiB. Hence, our study reflects that there are considerable differences between host and parasite Rpi enzyme and this would pave the way for future parasite specific drug design studies.

## Conclusion

In conclusion, the present study has highlighted the importance of conserved residues for *Ld*RpiB structure and function. The homology model were generated for wild type *Ld*RpiB and its mutants and various active site residues were identified of which the critically important residues involved in the substrate recognition phenomenon are Asp10, Thr71 and the positively charged residues at the surface are His11, Arg113, Arg137 and Arg141. The absence of substrate interaction with these residues leads to the inactivity of the enzyme. The current study can be summarized into five important findings i) Cys69, His102, His11, Asp45, Glu149 and Pro47 were residues identified in *Ld*RpiB enzyme being involved in substrate recognition, reaction catalysis and conformation, ii) His138 residue is involved only in substrate recognition and catalysis, iii) *In silico* studies indicated the presence of His11, His102, Cys69, His138 and Tyr46 residues in the active site pocket and Asp45, Glu149 and Pro47 out of the active site, iv) Apart from R5P as the natural substrate of *Ld*RpiB, D-ribose has emerged as another substrate candidate for this enzyme and v) interestingly, mutants which failed to bind to the natural R5P substrate showed varying specificity for other aldose substrates. The present findings can be further utilized for parasite specific drug design.

## Supporting Information

S1 Fig 2-Dimensional structures of substrate R5P (chain conformation; C-form), R5P (furanose conformation; F-form) and the product Ru5P. (TIF)

S2 Fig Structural alignment of template *Tc*RpiB (Blue) and the homology model of *Ld*RpiB (Green) with an RMSD of 0.087 Å. (TIF)

S3 Fig Molecular docking protocol validation by comparison of cocrystalized (green carbon) and redocked (cyan carbon) C-form of substrate R5P in: A) *Tc*RpiB (RMSD: 0.4787 Å) and B) *Ld*RpiB (RMSD: 0.5483 Å). (TIF)

S4 Fig 2D interaction diagrams for molecular recognition interaction of F-form and C-form of R5P in *Tc*RpiB (A and B) and *Ld*RpiB (C and D). (TIF)

S5 Fig Residue wise hydrogen bond occupancy for C-form of R5P in *Ld*RpiB wild type and various mutants. (TIF)

S6 Fig Residue wise hydrogen bond occupancy for F-form of R5P in *Ld*RpiB wild type and various mutants. (TIF)

S7 Fig Residue wise decomposition energy for F-form of R5P in *Ld*RpiB wild type and various mutants. (TIF)

S1 Table Oligonucleotide sequences for generating *Ld*RpiB and its mutants. (DOC)

S2 Table Molecular docking results for the F-form and C-form of R5P in the *Tc*RpiB crystal structure, *Ld*RpiB wild type homology model and various mutant *Ld*RpiB homology models. (DOCX)

S3 Table Average binding free energy results (last 4 ns) for enzyme-ligand complexes (*Tc*RpiB and *Ld*RpiB (wild type and various mutants) using C-form and F-form of substrate R5P along with its different energy components (GBSA). ^a^The meaning of different terms used in this table is as follows: VDW = van der Waals energy as calculated by the MM force field. EEL = electrostatic energy as calculated by the MM force field. EPB = the electrostatic contribution to the solvation free energy calculated by PB. ECAVITY = nonpolar contribution to the cavity solvation free energy calculated by PB. ΔGgas = total gas phase energy i.e. sum of van der Waals and electrostatic energy from MM. ΔGsolv = total solvation free energy i.e. sum of electrostatic and nonpolar contributions from solvation. ΔGbind = final estimated binding free energy calculated from the terms above (kcal/mol).
(DOC)
