# Supplementary figures and images for: Mutational and Structural Analysis of Conserved Residues in Ribose-5-Phosphate Isomerase B from Leishmania donovani: Role in Substrate Recognition and Conformational Stability
Source: PLoS One. 2016 Mar 8;11(3):e0150764. doi: 10.1371/journal.pone.0150764 (PMC4783025; doi:10.1371/journal.pone.0150764)

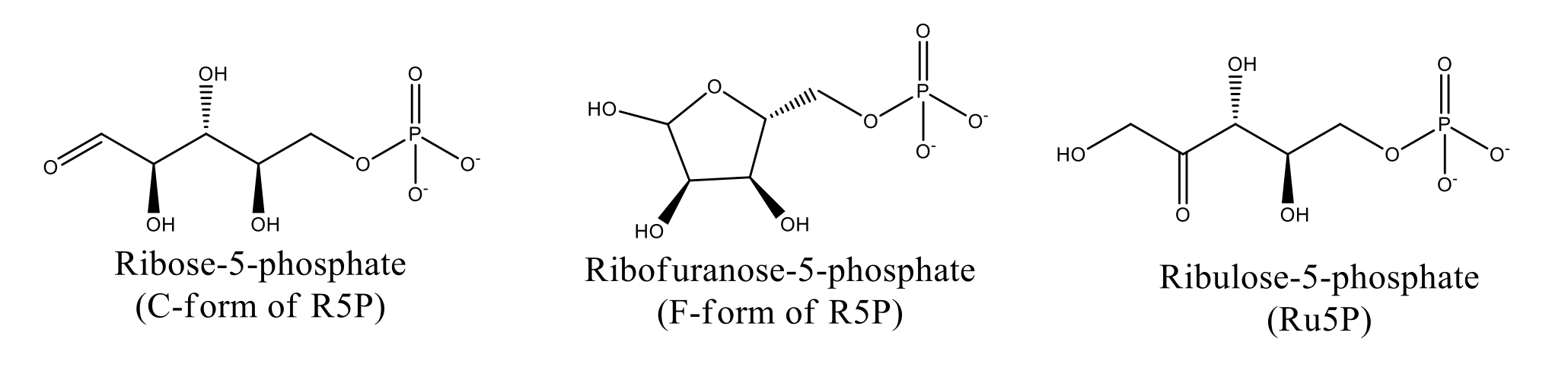

Supplement: S1 Fig — (TIF) [file pone.0150764.s001.tif]

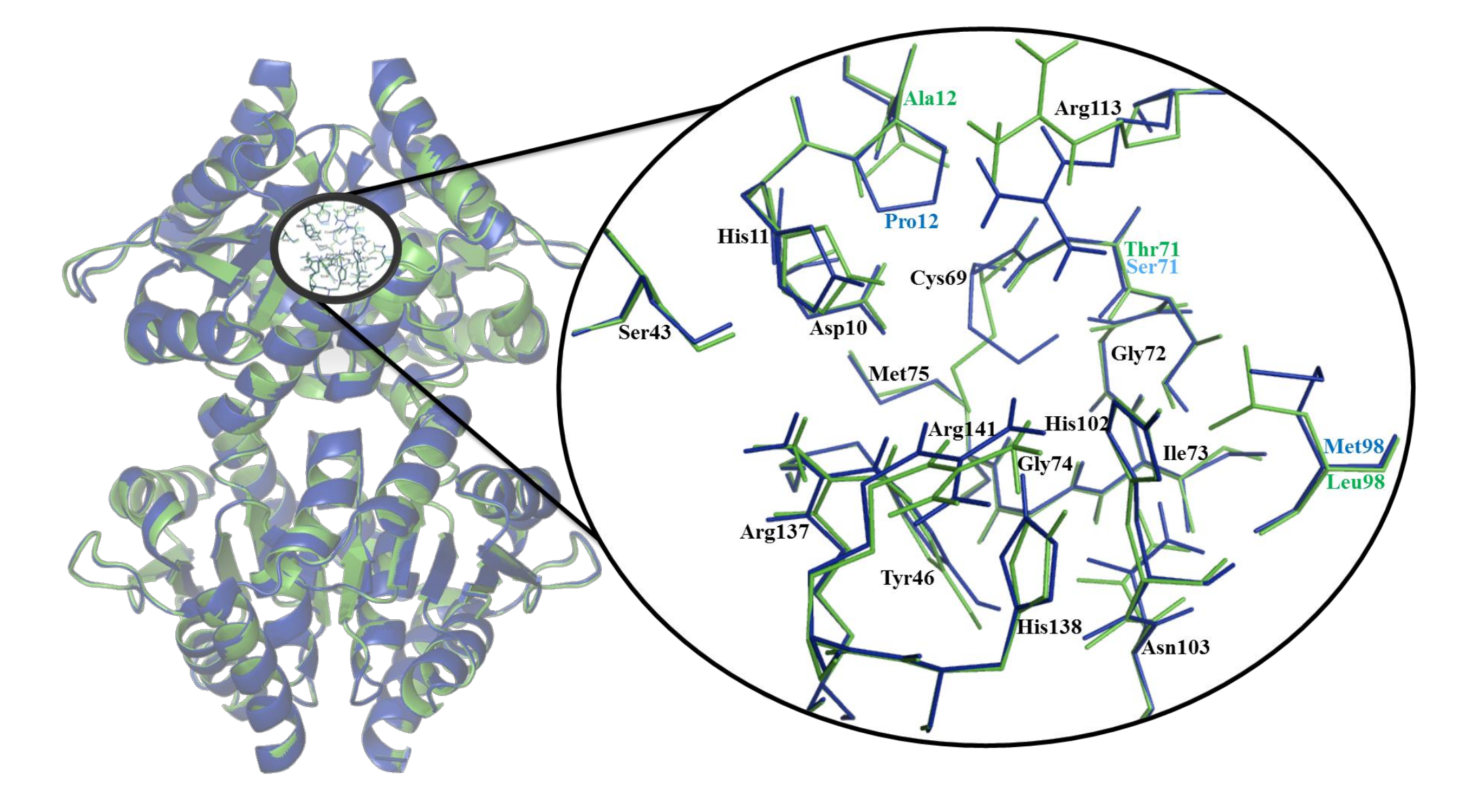

Supplement: S2 Fig — (TIF) [file pone.0150764.s002.tif]

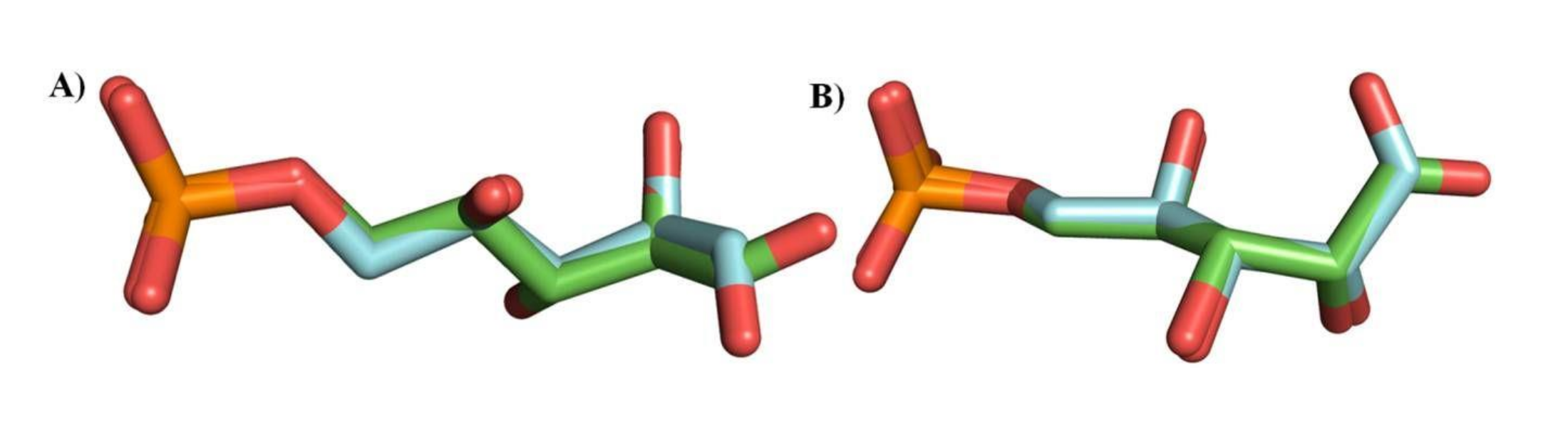

Supplement: S3 Fig — (TIF) [file pone.0150764.s003.tif]

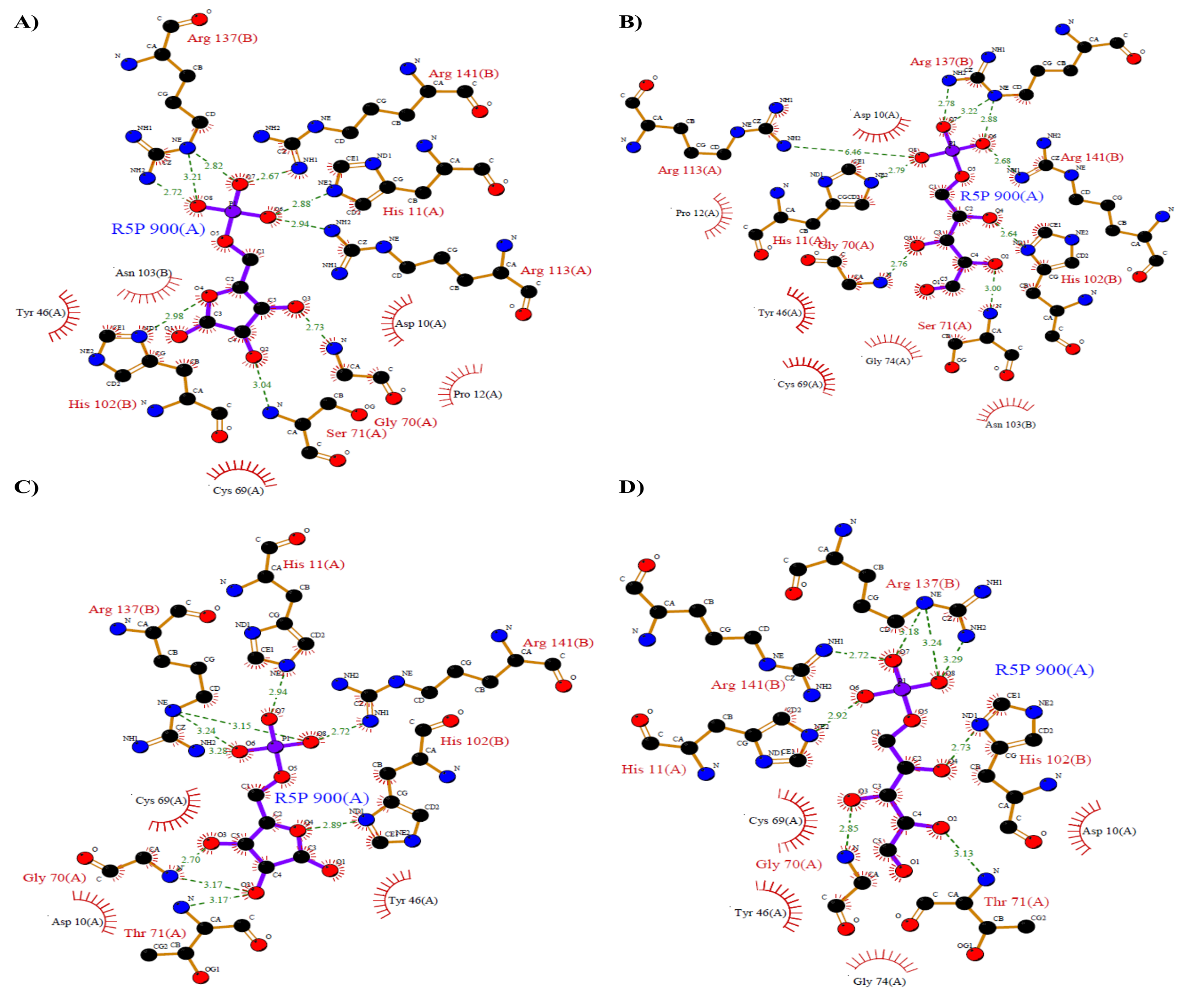

Supplement: S4 Fig — (TIF) [file pone.0150764.s004.tif]

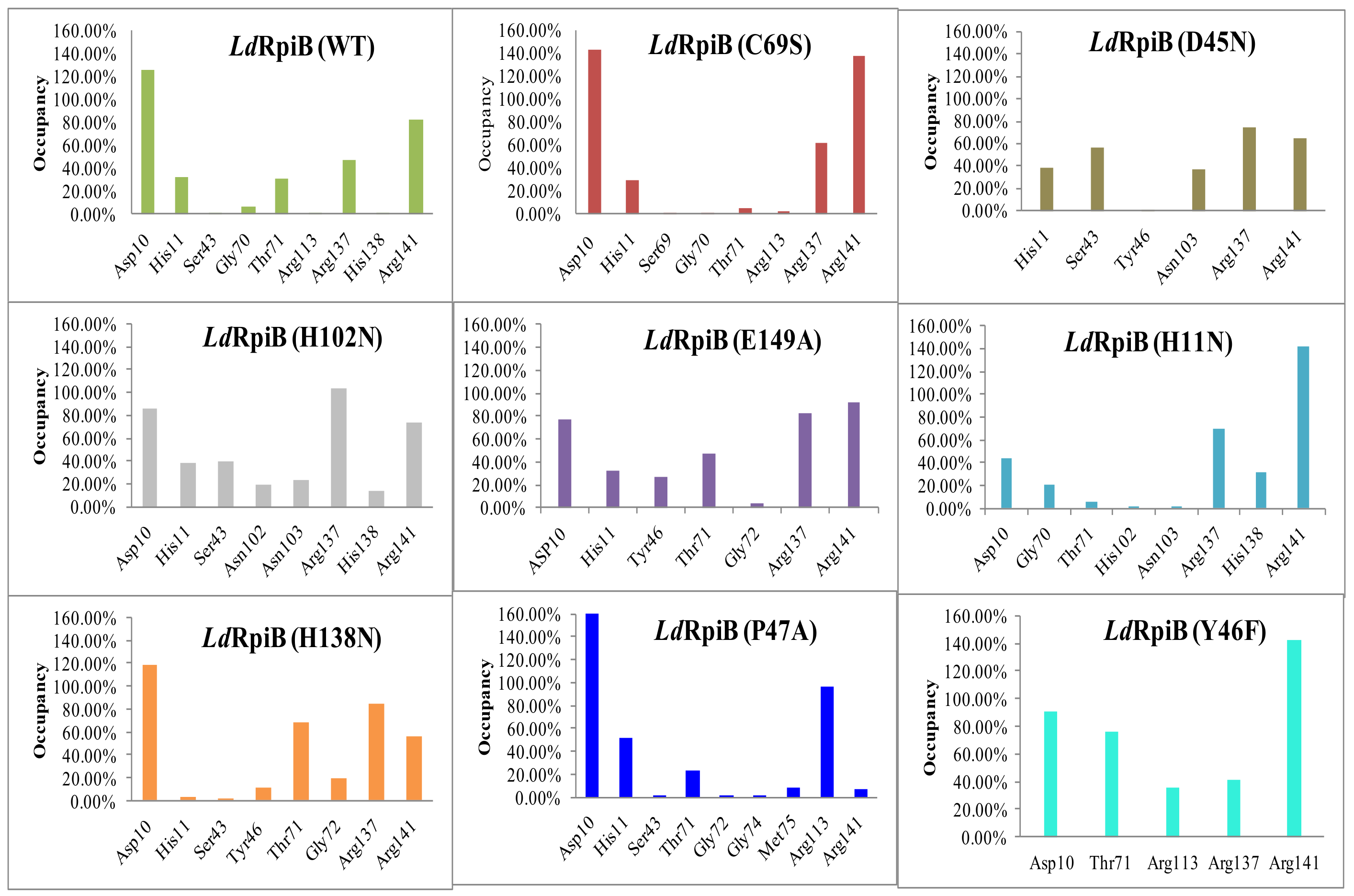

Supplement: S5 Fig — (TIF) [file pone.0150764.s005.tif]

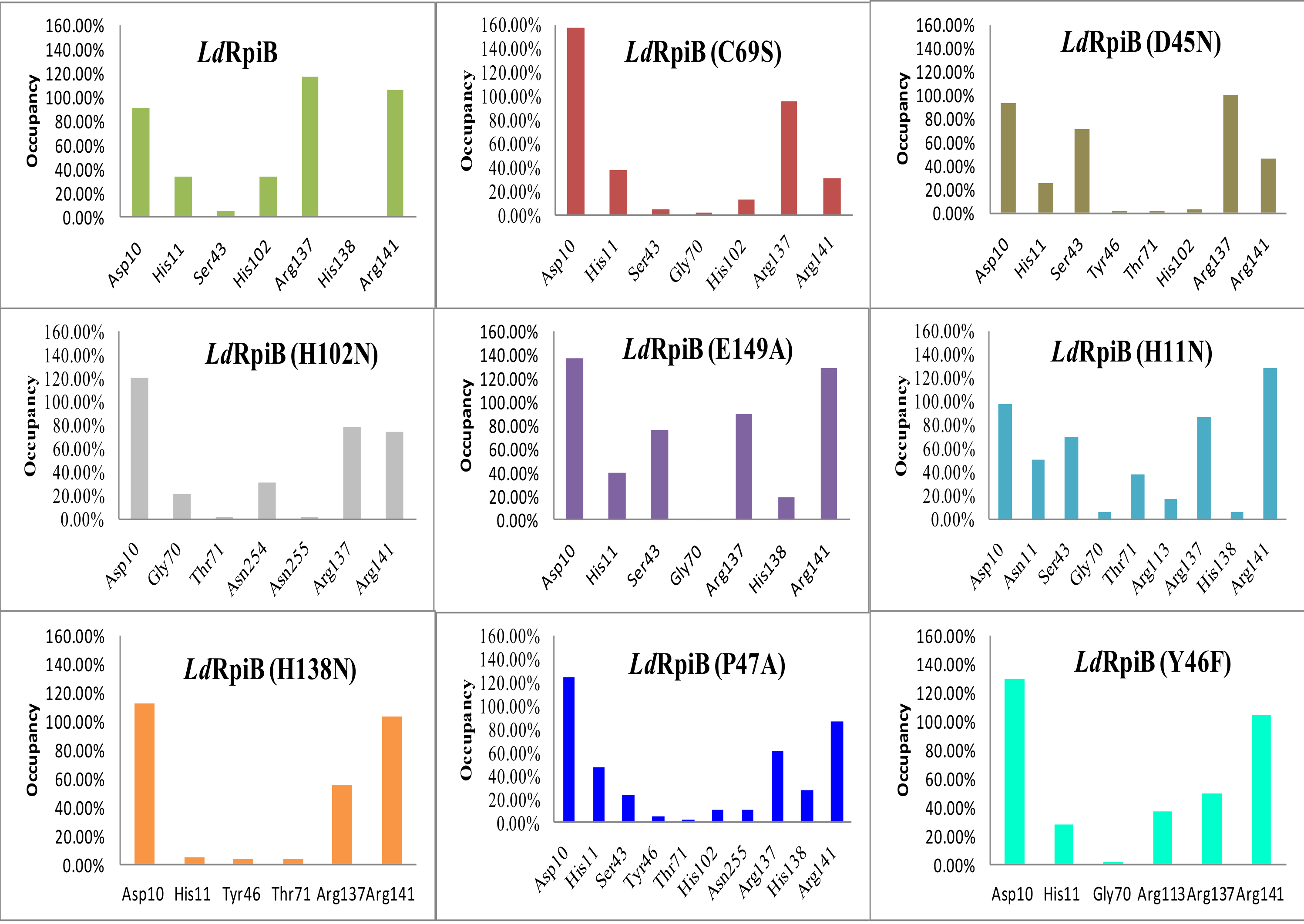

Supplement: S6 Fig — (TIF) [file pone.0150764.s006.tif]

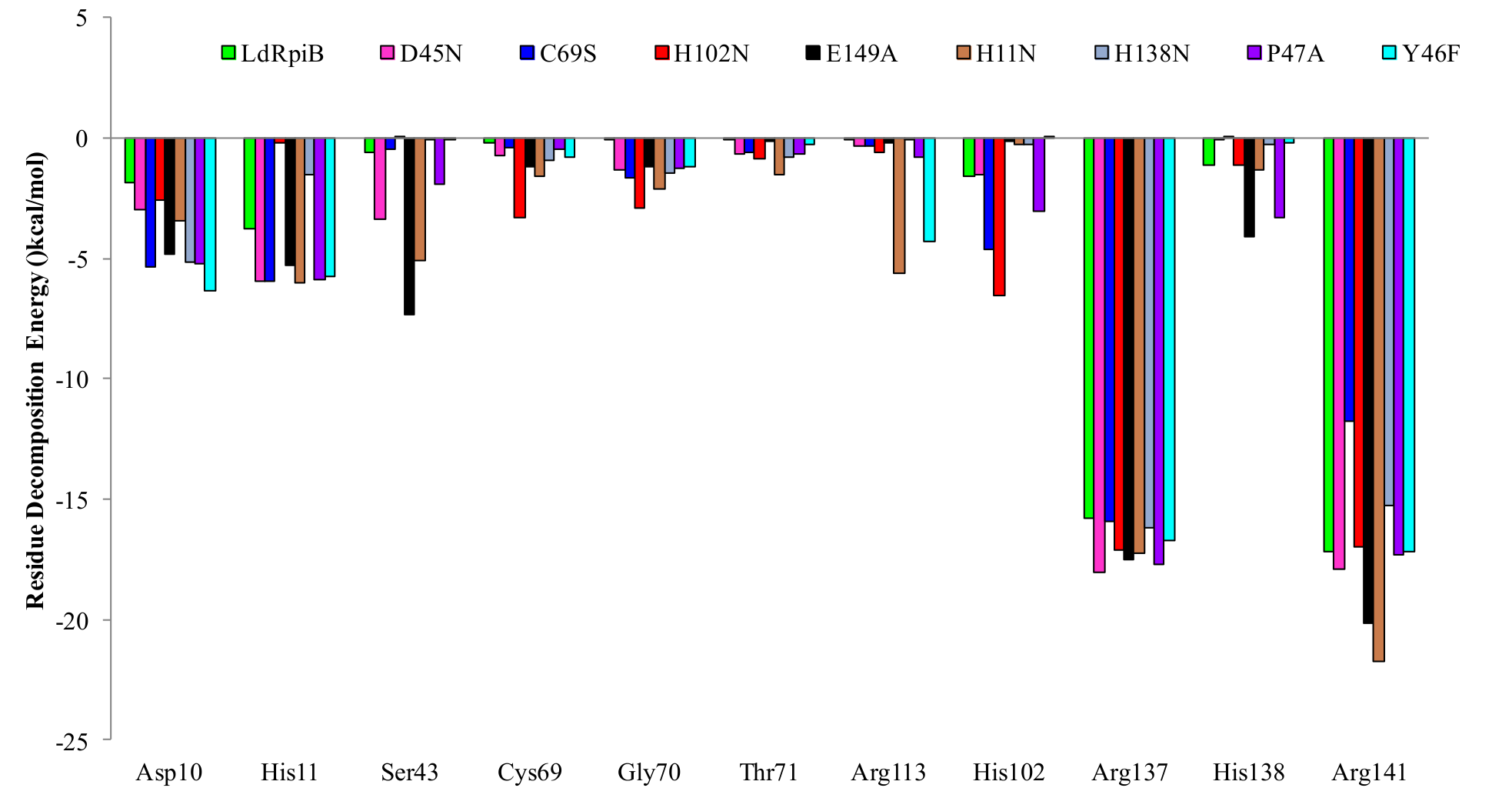

Supplement: S7 Fig — (TIF) [file pone.0150764.s007.tif]
