# Supplementary material for: Mutational and Structural Analysis of Conserved Residues in Ribose-5-Phosphate Isomerase B from Leishmania donovani: Role in Substrate Recognition and Conformational Stability
Source: PLoS One. 2016 Mar 8;11(3):e0150764. doi: 10.1371/journal.pone.0150764 (PMC4783025; doi:10.1371/journal.pone.0150764)
Supplement: S1 Table — (DOC) [file pone.0150764.s008.doc]

| **Primer Name Primer Sequences** |
| --- |
| RpiB Wild type Sense (P1) 5’ GGAATTC**CATATG**ATGCCGAAGCGTGTTGC 3’  Antisense (P2) 5’ CCG**CTCGAG**CTTTCCTTCCTCCTTAAGAC 3’  C69S Sense (P3) 5’ GGGCATCCTCGTC**A**GCGGCACCGGCATC 3’  Antisense (P4) 5’ GATGCCGGTGCCGC**T**GACGAGGATGCCC 3’  H102N Sense (P3) 5’GCTGAGCCGCCAG**A**ACAACAACGCCCAC 3’  Antisense (P4) 5’ GTGGGCGTTGTTGT**T**CTGGCGGCTCAGC 3’  H138N Sense (P3) 5’ GAGGAAGGGCGC**A**ACGGCAGCCGCC 3’  Antisense (P4) 5’ GGCGGCTGCCGT**T**GCGCCCTTCCTC 3’  E149A Sense (P3) 5’GATCACGGTGATCG**C**GGAGCAGCAGATG 3’  Antisense (P4) 5’CATCTGCTGCTCC**G**CGATCACCGTGATC 3’  D45N Sense (P3) 5’CCGACTCGTCTGTC**A**ACTACCCCGACTAC 3’  Antisense (P4) 5’ GTAGTCGGGGTAGT**T**GACAGACGAGTCGG 3’  Y46F Sense (P3) 5’ CGTCTGTCGACT**T**CCCCGACTACGC 3’  Antisense (P4) 5’ GCGTAGTCGGGG**A**AGTCGACAGACG 3’  P47A Sense (P3) 5’ GTCTGTCGACTAC**G**CCGACTACGCCGC 3’  Antisense (P4) 5’ GCGGCGTAGTCGG**C**GTAGTCGACAGAC 3’  H11N Sense 5’GGAATTC**CATATG**ATGCCGAAGCGTGTTGCTCTGG  GCTGCGAC**A**ACGCCGTCTACGCCGCACATCAGGAGATCATAGG 3’ |

S1 Oligonucleotide sequences for generating *Ld*RpiB and its mutants
