# Supplementary material for: Mutational and Structural Analysis of Conserved Residues in Ribose-5-Phosphate Isomerase B from Leishmania donovani: Role in Substrate Recognition and Conformational Stability
Source: PLoS One. 2016 Mar 8;11(3):e0150764. doi: 10.1371/journal.pone.0150764 (PMC4783025; doi:10.1371/journal.pone.0150764)
Supplement: S2 Table — (DOCX) [file pone.0150764.s009.docx]

| **Sl No** |  | **Glide gscore for two substrate conformations** | | | |
| --- | --- | --- | --- | --- | --- |
|  | **Mutants** | **Open** | **Hydrogen Bonding Residues** | **Ring** | **Hydrogen Bonding Residues** |
| 1 | *Tc*RpiB | -7.55 | Asp10, His11, Gly70, Ser71, His102, Arg137, Arg141 | -8.83 | Asp10, His11, Gly70, Ser71, Arg137, Arg141 |
| 2 | *Ld*RpiB (WT) | -6.16 | Asp10, His11, Gly70,His102, Arg113, Arg137, Arg141 | -7.60 | Asp10, His11, Gly70, Arg113, Arg137, Arg141 |
| 3 | *Ld*RpiB (C69S) | -6.30 | Asp10, His11,Ser69, Gly70, Gly74, His102, Arg113, Arg137, Arg141 | -7.55 | Asp10, His11, Gly70, Arg113, Arg137, Arg141 |
| 4 | *Ld*RpiB (D45N) | -6.94 | Asp10, His11, Gly74, His102, Arg137, Arg141 | -7.41 | Asp10, His11, Gly70, Arg113, Arg137, Arg141 |
| 5 | *Ld*RpiB (H11N) | -7.22 | Asp10, Gly70,His102, Arg113, Arg137, Arg141 | -5.94 | Asp10, Gly70, Arg113, Arg137, Arg141 |
| 6 | *Ld*RpiB (H102N) | -6.08 | Asp10, His11, Gly70,Asn102, Arg113, Arg137, Arg141 | -7.53 | Asp10, His11, Gly70, Arg113, Arg137, Arg141 |
| 7 | *Ld*RpiB (H138N) | -6.08 | Asp10, His11, Gly70,His102, Arg113, Arg137, Arg141 | -7.28 | Asp10, His11, Gly70, Arg113, Arg137, Arg141 |
| 8 | *Ld*RpiB (E149A) | -6.16 | Asp10, His11, Gly70,His102, Arg113, Arg137, Arg141 | -7.63 | Asp10, His11, Gly70, Arg113, Arg137, Arg141 |
| 9 | *Ld*RpiB (P47A) | -6.16 | Asp10, His11, Gly70,His102, Arg113, Arg137, Arg141 | -7.39 | Asp10, His11, Gly70, Arg113, Arg137, Arg141 |
| 10 | *Ld*RpiB (Y46F) | -5.88 | Asp10, His11, Gly70,His102, Arg113, Arg137, Arg141 | -7.44 | Asp10, His11, Gly70, Arg113, Arg137, Arg141 |

S2 Molecular docking results for the F-form and C-form of R5P in the *Tc*RpiB crystal structure, *Ld*RpiB wild type homology model and various mutant *Ld*RpiB homology models
