# Supplementary material for: Mutational and Structural Analysis of Conserved Residues in Ribose-5-Phosphate Isomerase B from Leishmania donovani: Role in Substrate Recognition and Conformational Stability
Source: PLoS One. 2016 Mar 8;11(3):e0150764. doi: 10.1371/journal.pone.0150764 (PMC4783025; doi:10.1371/journal.pone.0150764)
Supplement: S3 Table — aThe meaning of different terms used in this table is as follows: VDW = van der Waals energy as calculated by the MM force field. EEL = electrostatic energy as calculated by the MM force field. EPB = the electrostatic contribution to the solvation free energy calculated by PB. ECAVITY = nonpolar contribution to the cavity solvation free energy calculated by PB. ΔGgas = total gas phase energy i.e. sum of van der Waals and electrostatic energy from MM. ΔGsolv = total solvation free energy i.e. sum of electrostatic and nonpolar contributions from solvation. ΔGbind = final estimated binding free energy calculated from the terms above (kcal/mol). (DOC) [file pone.0150764.s010.doc]

| **Enzyme/ Mutants** | **Substrate Conformation** | **VDW** | **EEL** | **EGB** | **ESURF** | **ΔG_gas_** | **ΔG_solv_** | **ΔG_bind_** |
| --- | --- | --- | --- | --- | --- | --- | --- | --- |
| ***Tc*RpiB** | Open | -13.93 | -195.50 | 168.46 | -3.41 | -209.43 | 165.05 | -44.38 ± 7.07 |
| ***Ld*RpiB** | Open | -12.22 | -136.92 | 112.09 | -3.35 | -149.14 | 108.74 | -40.39 ± 5.09 |
| **C69S** | Open | -11.51 | -157.26 | 131.34 | -3.45 | -168.78 | 127.89 | -40.88 ± 5.38 |
| **D45N** | Open | -12.63 | -178.79 | 169.99 | -3.52 | -191.42 | 166.47 | -24.95 ± 4.80 |
| **E149A** | Open | -10.87 | -234.98 | 205.66 | -3.10 | -245.85 | 202.56 | -43.29 ± 5.23 |
| **H102N** | Open | -10.59 | -128.05 | 99.65 | -3.50 | -138.65 | 96.16 | -42.49 ± 7.47 |
| **H11N** | Open | -10.99 | -151.04 | 125.05 | -3.24 | -162.04 | 121.81 | -40.22 ± 9.36 |
| **H138N** | Open | -13.06 | -118.03 | 96.57 | -3.36 | -131.09 | 93.21 | -37.88 ± 9.69 |
| **P47A** | Open | -18.23 | -85.84 | 73.62 | -3.30 | -104.07 | 70.32 | -33.76 ± 6.54 |
| **Y46F** | Open | -11.78 | -156.94 | 127.36 | -3.27 | -168.71 | 124.09 | -44.62 ± 4.86 |
| ***Tc*RpiB** | Ring | -7.89 | -222.65 | 197.61 | -2.59 | -230.54 | 195.02 | -35.53 ± 9.87 |
| ***Ld*RpiB** | Ring | -6.61 | -127.56 | 105.37 | -2.98 | -134.18 | 102.39 | -31.78 ± 7.15 |
| **C69S** | Ring | -12.45 | -159.70 | 142.04 | -3.44 | -172.15 | 138.60 | -33.56 ± 4.40 |
| **D45N** | Ring | -8.98 | -240.27 | 213.64 | -3.32 | -249.26 | 210.32 | -38.94 ± 5.75 |
| **E149A** | Ring | -7.46 | -231.89 | 183.71 | -3.39 | -239.35 | 180.32 | -59.03 ± 5.53 |
| **H102N** | Ring | -11.51 | -170.14 | 138.02 | -3.59 | -181.65 | 134.44 | -47.21 ± 6.36 |
| **H11N** | Ring | -13.32 | -196.93 | 145.21 | -3.53 | -210.25 | 141.68 | -68.57 ± 10.83 |
| **H138N** | Ring | -10.13 | -92.74 | 74.16 | -3.32 | -102.87 | 70.84 | -32.03 ± 4.26 |
| **P47A** | Ring | -12.49 | -169.62 | 131.36 | -3.47 | -182.11 | 127.89 | -54.22 ± 8.27 |
| **Y46F** | Ring | -5.47 | -165.94 | 124.78 | -3.01 | -171.41 | 121.77 | -49.65 ± 5.39 |

S3 Average binding free energy results (last 4 ns) for enzyme-ligand complexes (*Tc*RpiB and *Ld*RpiB (wild type and various mutants) using C-form and F-form of substrate R5P along with its different energy components (GBSA)
